# Mutations in PBP2 from ceftriaxone-resistant *Neisseria gonorrhoeae* alter the dynamics of the β3–β4 loop to favor a low-affinity drug-binding state

**DOI:** 10.1016/j.jbc.2021.101188

**Published:** 2021-09-13

**Authors:** Benjamin A. Fenton, Joshua Tomberg, Carly A. Sciandra, Robert A. Nicholas, Christopher Davies, Pei Zhou

**Affiliations:** 1 Department of Biochemistry, Duke University School of Medicine, Durham, North Carolina, USA; 2 Departments of Pharmacology and Microbiology and Immunology, University of North Carolina at Chapel Hill, Chapel Hill, North Carolina, USA; 3 Department of Biochemistry and Molecular Biology, Medical University of South Carolina, Charleston, South Carolina, USA

**Keywords:** PBP2, *Neisseria gonorrhoeae*, beta-lactam, conformational dynamics, antibiotic resistance, BTFA, bromotrifluoroacetone, ESC, extended-spectrum cephalosporin, ITC, isothermal titration calorimetry, KTG, Lys^497^Thr^498^Gly^499^, PBP2, penicillin-binding protein 2, PDB, Protein Data Bank, TPase, transpeptidase

## Abstract

Resistance to the extended-spectrum cephalosporin ceftriaxone in the pathogenic bacteria *Neisseria gonorrhoeae* is conferred by mutations in penicillin-binding protein 2 (PBP2), the lethal target of the antibiotic, but how these mutations exert their effect at the molecular level is unclear. Using solution NMR, X-ray crystallography, and isothermal titration calorimetry, we report that WT PBP2 exchanges dynamically between a low-affinity state with an extended β3–β4 loop conformation and a high-affinity state with an inward β3–β4 loop conformation. Histidine-514, which is located at the boundary of the β4 strand, plays an important role during the exchange between these two conformational states. We also find that mutations present in PBP2 from H041, a ceftriaxone-resistant strain of *N. gonorrhoeae*, increase resistance to ceftriaxone by destabilizing the inward β3–β4 loop conformation or stabilizing the extended β3–β4 loop conformation to favor the low-affinity drug-binding state. These observations reveal a unique mechanism for ceftriaxone resistance, whereby mutations in PBP2 lower the proportion of target molecules in the high-affinity drug-binding state and thus reduce inhibition at lower drug concentrations.

*Neisseria gonorrhoeae* is the causative agent of the sexually transmitted infection gonorrhea, with nearly 80 million cases worldwide each year (1). Without antibiotic treatment, infections persist as a chronic disease and can cause serious sequelae, including pelvic inflammatory disease, infertility, arthritis, and disseminated infections (2). For many years, *N. gonorrhoeae* was treated with a single dose of penicillin, and more recently, ceftriaxone. In 2012, the emergence of several high-level ceftriaxone-resistant strains led the Centers for Disease Control and Prevention to change its recommended treatment for gonorrhea from monotherapy to dual therapy with ceftriaxone and azithromycin (3, 4, 5). However, treatment failures have been reported for both agents, and in 2018, a strain with high-level resistance to both ceftriaxone and azithromycin was identified (6, 7). Concern about azithromycin resistance led the Centers for Disease Control and Prevention recently to drop the recommendation of dual therapy in favor of an increased dose (500 mg) of ceftriaxone alone (8). Both penicillin and ceftriaxone inhibit cell wall biosynthesis in *N. gonorrhoeae* by targeting penicillin-binding protein 2 (PBP2).

PBP2 is an essential peptidoglycan transpeptidase (TPase) that crosslinks the peptide chains from adjacent peptidoglycan strands during cell-wall synthesis (9). β-lactam antibiotics, including the extended-spectrum cephalosporin (ESC) ceftriaxone, are analogs of the d-Ala-d-Ala C terminus of the peptidoglycan substrate and as such target PBP2 by binding to and reacting with the active-site serine nucleophile (Ser310 in *N. gonorrhoeae* PBP2) to form a covalently acylated complex (10, 11). The acylation reaction (Equation 1) proceeds first through formation of a noncovalent complex with the β-lactam (defined by the equilibrium constant, Ks), which is then attacked by the serine nucleophile to form a covalent acyl-enzyme complex (*k*_2_). For PBPs, hydrolysis of the acyl-enzyme (*k*_3_) is very slow compared with its formation, and the enzyme is essentially irreversibly inactivated. The acylation of PBPs by β-lactam antibiotics is therefore defined by a second-order rate constant, *k*_2_/Ks (M^−1^ s^−1^), which reflects both the noncovalent binding affinity (Ks) and the first-order acylation rate (*k*_2_):

(1) $$E+S\overset{\text{Ks}}{↔}E\cdot S\overset{k_{2}}{\to }E-S\overset{k_{3}}{\to }E+P$$

The emergence of resistance to penicillin and ceftriaxone in *N. gonorrhoeae* occurs primarily *via* the acquisition of mutant alleles of the *penA* gene encoding PBP2 (12). These alleles are referred to as mosaic because they arise through multiple homologous recombination events with DNA released by commensal *Neisseria* species. PBP2 from the high-level ceftriaxone-resistant strain, H041, contains 61 mutations compared with PBP2 from the antibiotic-susceptible strain, FA19 (13, 14). Determining how these mutations lower the *k*_2_/Ks of ceftriaxone for PBP2 by over 10,000-fold while still preserving essential TPase activity is fundamental for understanding the evolution of antibiotic resistance.

Toward this goal, we have identified a subset of these mutations that, when incorporated into the *penA* gene from FA19, confer ∼80% of the increase in minimum inhibitory concentration for ceftriaxone relative to that of the *penA* gene from H041 (*penA41*) (15, 16). We recently reported the structures of *apo* and ceftriaxone-acylated PBP2 at high resolution and have detailed conformational changes in β3 and the β3–β4 loop involved in antibiotic binding and acylation (17). Intriguingly, although present in the active site region, most of the mutations conferring resistance are not in direct contact with ceftriaxone in the crystal structure of acylated PBP2 (17, 18). We have proposed that these mutations alter the binding and acylation kinetics of PBP2 with ceftriaxone by restricting protein dynamics (18).

To understand further the structural and biochemical mechanisms by which these mutations lower the acylation rates of β-lactam antibiotics, we utilized a combination of solution ^19^F NMR, X-ray crystallography, and biochemical approaches to investigate PBP2. We report that the β3–β4 loop in the TPase domain of WT PBP2, which is known to adopt markedly different conformations in the *apo versus* acylated crystal structures (17), samples two major conformational states in solution. Substitutions of WT PBP2 residues with mutations in H041 that confer ceftriaxone resistance alter the conformational landscape of PBP2 by destabilizing the high-affinity state containing the inward conformation of the β3–β4 loop and stabilizing a low-affinity conformation containing an extended β3–β4 loop conformation, thereby restricting access to the inward conformation required for high-affinity drug binding. Our combined solution NMR and crystallographic analyses of PBP2 and its preacylation drug complexes further support the notion that mutations in PBP2 from ceftriaxone-resistant strains of *N. gonorrhoeae* confer antibiotic resistance by hindering conformational changes required to form a productive drug-binding state (18).

## Results

### ^19^F NMR reveals the presence of two major conformational states in WT PBP2

PBP2 is comprised of an N-terminal membrane anchor, a putative pedestal domain, and a C-terminal catalytic TPase/β-lactam–binding domain (19). To make the protein more amenable to structural studies by X-ray crystallography and NMR, we previously generated a construct of PBP2 comprising only the TPase domain, engineered by deletion of the N-terminal domain and a protruding loop comprising residues 283 to 297. This protein, referred to hereafter as tPBP2, where t stands for “truncated,” displays identical acylation kinetics as the full-length protein (20).

**Figure 1:**
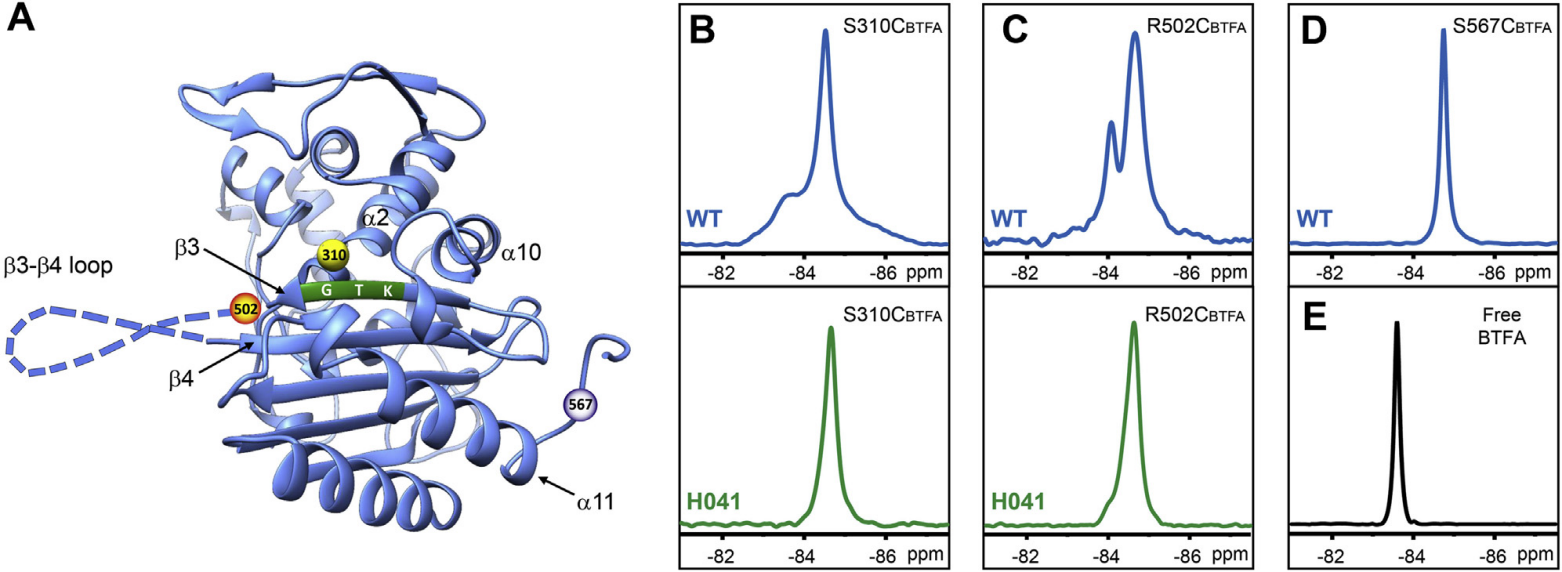
**Conformational dynamics of tPBP2.***A*, ribbon representation of the transpeptidase domain of tPBP2^WT^ (Protein Data Bank: 3EQU). The locations of the catalytic residue Ser310 (*yellow sphere*), Arg502 (*orange sphere*), Ser567 (*gray sphere*), and the KTG motif (*green ribbon*) are highlighted. The β3–β4 loop is illustrated as a *dashed loop*. *B*, ^19^F NMR spectra of tPBP2^WT^–S310C_BTFA_ (*upper panel*) and tPBP2^H041^–S310C_BTFA_ (*lower panel*). *C*, ^19^F NMR spectrum of tPBP2^WT^–R502C_BTFA_ (*upper panel*) and tPBP2^H041^–R502C_BTFA_ (*lower panel*). *D*, ^19^F NMR spectrum of tPBP2^WT^–S567C_BTFA_ (negative control). *E*, ^19^F NMR spectrum of free BTFA. NMR spectra in panels (*B*–*E*) were collected at pH 7.5. BTFA, bromotrifluoroacetone; KTG, Lys^497^Thr^498^Gly^499^; tPBP2, truncated construct of penicillin-binding protein 2.

The TPase domain of PBP2 contains a central five-stranded antiparallel β-sheet surrounded by α-helices on both sides (Fig. 1*A*). The serine nucleophile, Ser310, located at the N-terminal end of the α2 helix, is spatially adjacent to the β3 strand of the central β-sheet. β3 contains the conserved Lys^497^Thr^498^Gly^499^ (KTG) motif that twists during β-lactam antibiotic binding to form the oxyanion hole for acylation (17). The β3 strand continues from the KTG motif and extends away from the active site before looping back to β4. The β3–β4 loop contains two mutations, F504L and N512Y, that are associated with β-lactam resistance (15, 19).

To gain mechanistic insights into how mutations that surround the active site confer resistance, we employed solution ^19^F NMR to investigate the dynamics of PBP2 from the ceftriaxone-susceptible strain, FA19 (tPBP2^WT^) and the ceftriaxone-resistant strain, H041 (tPBP2^H041^). Neither of these tPBP2 variants contains a native cysteine, allowing site-specific incorporation of a ^19^F probe at selected locations *via* site-directed mutagenesis to introduce a cysteine, followed by bromotrifluoroacetone (BTFA) labeling (21). The result is a cysteine side chain alkylated with a trifluoroacetone group, which we denote with a subscript “_BTFA_” (22, 23).

We first introduced the ^19^F probe at the active site of WT PBP2 *via* a S310C mutation (tPBP2^WT^–S310C_BTFA_). Our 1D ^19^F NMR measurements revealed two signals (Fig. 1*B*, *upper panel*) indicative of two conformations in the protein, with a predominant upfield signal centered at −84.5 ppm and a smaller downfield signal at −83.6 ppm. We also made an R502C mutant (tPBP2^WT^–R502C_BTFA_), which is located on the C-terminal end of the KTG active site motif on the β3 strand, and a similar spectrum was observed (Fig. 1*C*, *upper panel*), with the upfield ^19^F signal at −84.7 ppm and the downfield ^19^F signal at −84.1 ppm. As a control, we labeled tPBP2^WT^–S567C, which is located in the C-terminal tail of the protein at a position distant from the active site (Fig. 1*A*), and the ^19^F NMR spectra of tPBP2^WT^–S567C_BTFA_ displayed a single and sharp ^19^F signal at −84.8 ppm consistent with a single conformation of the protein in this region (Fig. 1*D*). Because the observed dual signals of tPBP2^WT^–S310C_BTFA_ and tPBP2^WT^–R502C_BTFA_ either have distinct chemical shifts or have very different line shapes from the sharp peak at −83.5 ppm for free BTFA (Fig. 1*E*), they are unlikely to arise from any free BTFA remaining in the samples (free BFTA was removed by buffer exchange and gel filtration; see Experimental procedures section). Taken together, these observations demonstrate that *apo* tPBP2^WT^ exists in two distinct conformational states in the active site region.

### PBP2^H041^ exhibits only a single conformational state

The two conformations of tPBP2^WT^ suggested by ^19^F NMR likely represent different conformations of the β3–β4 loop, which undergoes a large conformational shift upon acylation in crystal structures of tPBP2^WT^ (18). To determine whether these two conformational states are also present in tPBP2^H041^, we recorded the solution NMR spectra of tPBP2^H041^ using the same probes used for tPBP2^WT^. Only a single ^19^F signal was observed in tPBP2^H041^, regardless of whether the probe was positioned at 310 or 502 (Fig. 1, *B* and *C*, *lower panels*). Furthermore, in both cases, the single ^19^F signals of tPBP2^H041^ (−84.6 ppm for tPBP2^H041^–S310C_BTFA_ and −84.7 ppm for tPBP2^H041^–R502C_BTFA_) overlapped with the upfield ^19^F signal of tPBP2^WT^. This suggests that the β3–β4 loop in tPBP2^H041^ is restricted to one of the two conformations observed in the WT enzyme, and congruent with its role in cephalosporin resistance, represents a conformation with a reduced capacity for acylation. These data are consistent with the crystal structures of *apo* and acylated tPBP2^H041^, which show that, in contrast to tPBP2^WT^, the β3–β4 loop in tPBP2^H041^ remains in an extended and outward conformation and does not move when acylated by ceftriaxone (18).

**Figure S1:**
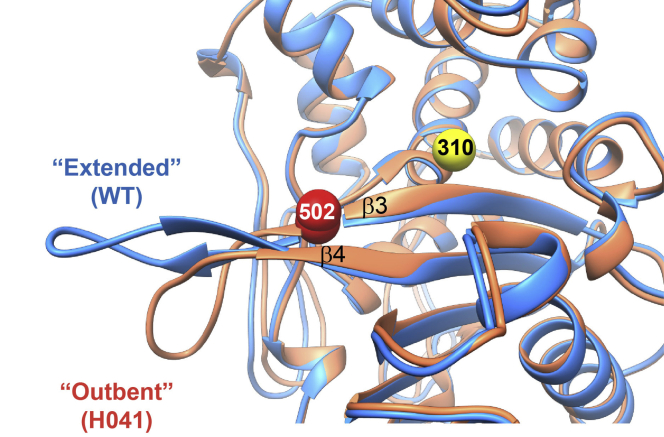
**Structural overlay of the extended and “outbent” β3–β4 loop conformations.** The *ribbon* structures of tPBP2 at pH 9.3 (PDB ID: 6P53; *blue*) and tPBP2-H041 at pH 9.3 (PDB ID: 6VBC; *orange*) are aligned, showing differences in the distal end of the β3–β4 loop. The locations of the active site residues Ser310 and Arg502 are labeled with *yellow* and *red spheres*, respectively, in both structures.

In the previous crystal structures of tPBP2^WT^ and tPBP2^H041^, the extended forms of the β3–β4 loop are different, where the loop in tPBP2^H041^ occupies a position relatively further away from the active site in a conformation we termed “outbent.” We note that the extended conformations of tPBP2^WT^ and tPBP2^H041^ instead appear at the same positions in ^19^F NMR spectra for the S310C_BTFA_ and R502C_BTFA_ proteins (Fig. 1, *B* and *C*). This is either because the extended conformation in the crystal structures is influenced by crystal packing interactions (18) or because the local environments of R502C_BTFA_ and S310C_BTFA_ of these two conformations when the proteins are in solution are similar (Fig. S1). Thus, additional ^19^F probes might be necessary to differentiate the outbent conformation from the extended conformation of the β3–β4 loop in future studies.

### H514 plays a critical role in conformational flexibility

**Figure 2:**
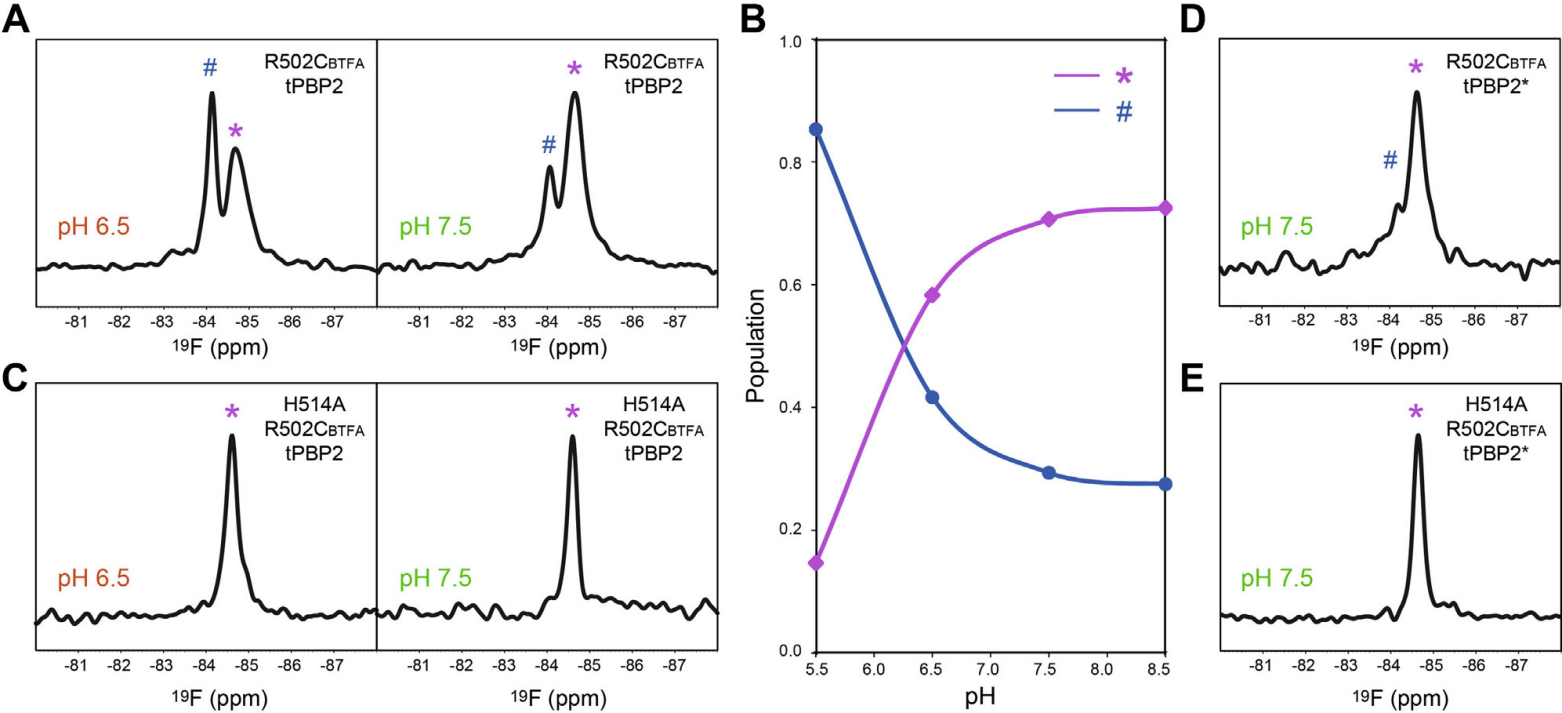
**His514 plays an important role in pH-dependent conformational changes of tPBP2.***A*, ^19^F NMR spectra of tPBP2–R502C_BTFA_ ats pH 6.5 and 7.5. The upfield and downfield peaks are labeled with the asterisk (∗) and pound (#) signs, respectively. *B*, pH-dependent population shift of the tPBP2 conformations represented by the two ^19^F signals. *C*, ^19^F NMR spectra of tPBP2–H514A–R502C_BTFA_ at pH 6.5 and 7.5. *D*, the ^19^F NMR spectrum of tPBP2∗–R502C_BTFA_ at pH 7.5. *E*, the ^19^F NMR spectrum of tPBP2∗–H514A–R502C_BTFA_ at pH 7.5. tPBP2, truncated construct of penicillin-binding protein 2.

**Figure S2:**
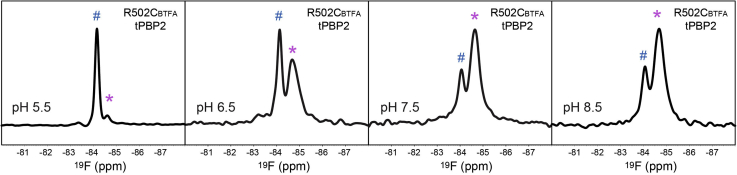
^**19**^**F NMR spectra of R502C**_**BTFA**_**tPBP2 at different pH values.** The locations of the upfield and downfield signals are indicated by the *asterisk* (∗) and *pound* (#) signs, respectively. The ^19^F NMR spectra of pH 6.5 and 7.5 (Fig. 2*A*) are included here to illustrate the population changes of the upfield and downfield signals across the pH range from 5.5 to 8.5.

Since Arg502 is positioned at the junction of the β3 strand and the β3–β4 loop, we focused on tPBP2–R502C_BFTA_ to probe for conformational changes in the β3–β4 loop region. As pH is a common factor that could affect the conformational equilibrium, we investigated whether it could affect the populations of the two observed states. Compared with the two peaks of tPBP2–R502C_BTFA_ observed at neutral pH (pH = 7.5), the downfield ^19^F signal became more populated at lower pH (pH = 6.5) (Fig. 2*A*). The relative populations of these two states changed with pH: the population of the downfield signal gradually reduced from 85% at pH 5.5 to 28% at pH 8.5, whereas the upfield signal correspondingly increased from 15 to 72% (Fig. 2*B*; raw spectra shown in Fig. S2). At pH ranges below 5.5 or above 8.5, the protein sample became unstable during NMR experiments, likely reflecting protein aggregation or denaturation. Taken together, the NMR experiments indicate the presence of a pH-sensitive and titratable residue near R502C with an estimated p*K*a between 6.0 and 6.5 that can alter the conformation of the β3–β4 loop.

As His514 is the only titratable residue in the β3–β4 loop within the range of pH values tested, we investigated whether the pH-dependent phenomenon was altered with a H514A mutant. Consistent with this idea, we observed a single ^19^F signal in tPBP2–H514A–R502C_BTFA_ (Fig. 2*C*) and, in contrast to tPBP2–R502C_BTFA_, no changes in the spectrum were observed at pH 6.5 and 7.5 (Fig. 2*C*). These data suggest that His514 is required for the pH-dependent conformational change of β3–β4. Since the single ^19^F NMR signal of tPBP2–H514A–R502C_BTFA_ (−84.6 ppm; Fig. 2*C*) is located at the same upfield position as the single peak observed with tPBP2^H041^–R502C_BTFA_ (–84.7 ppm; Fig. 1*C*, *bottom panel*), these observations support the idea that the tPBP2–H514A mutant adopts a similar conformation as the H041 mutant in solution and one of the two conformations sampled by the WT enzyme.

In order to provide a direct correlation of the dynamics observed by NMR with affinity for ceftriaxone separate from acylation, and elucidate whether pH changes and the H514A mutant have a consequence on the PBP2–ceftriaxone interaction, we introduced a catalytically inactive S310A mutant of tPBP2^WT^ (tPBP2^WT^–S310A, hereafter referred to as tPBP2∗). The same patterns were observed in the ^19^F spectrum for tPBP2∗–R502C_BTFA_ (Fig. 2*D*) and tPBP2∗–R502C_BTFA_–H154A (Fig. 2*E*) at pH 7.5 as for the catalytically active enzyme (Fig. 2, *A* and *C*), showing that mutation of the active site S310A does not have a significant effect on the two conformational states.

### Crystal structures of tPBP2∗ and tPBP2∗–H514A

To understand how pH affects the structure of tPBP2, we determined the crystal structures of tPBP2∗ at pH 7.5 and 9.5 and of tPBP2∗–H514A at pH 7.5 (Table 1). All structures were obtained in the same P2_1_ crystal system, with two molecules present in each asymmetric unit, as previously reported for the WT enzyme (17, 19). Hence, any conformational differences observed in these crystals should reflect a genuine structural change, rather than result from different packing environments.

**Table 1:** Randomization of His514 in *penA41* reveals amino acid substitutions capable of supporting cell growth and viability

Experiment	Results
Replacement of His514 codon CAC with NNN	• 10 CATs (His) • 11 CACs (His) • 1 CTG (Leu) • 1 TGG (Trp) • 6 *penA41*/*penAwt* hybrid sequences that recombined before the His514 codon
Replacement of His514 codon CAC with (A/G/T)NN (no C at first position in codon)	• 3 TTGs (Leu) • 13 *penA41/penAwt* hybrid sequences that recombined before the His514 codon
Replacement of His514 codon CAC with CN(A/G) (no T or C at last position in codon)	• 5 CAGs (Gln) • 1 CAA (Gln) • 2 CTAs (Leu) • 1 CTG (Leu) • 3 *penA41/penAwt* hybrid sequences that recombined before the His514 codon

**Figure 3:**
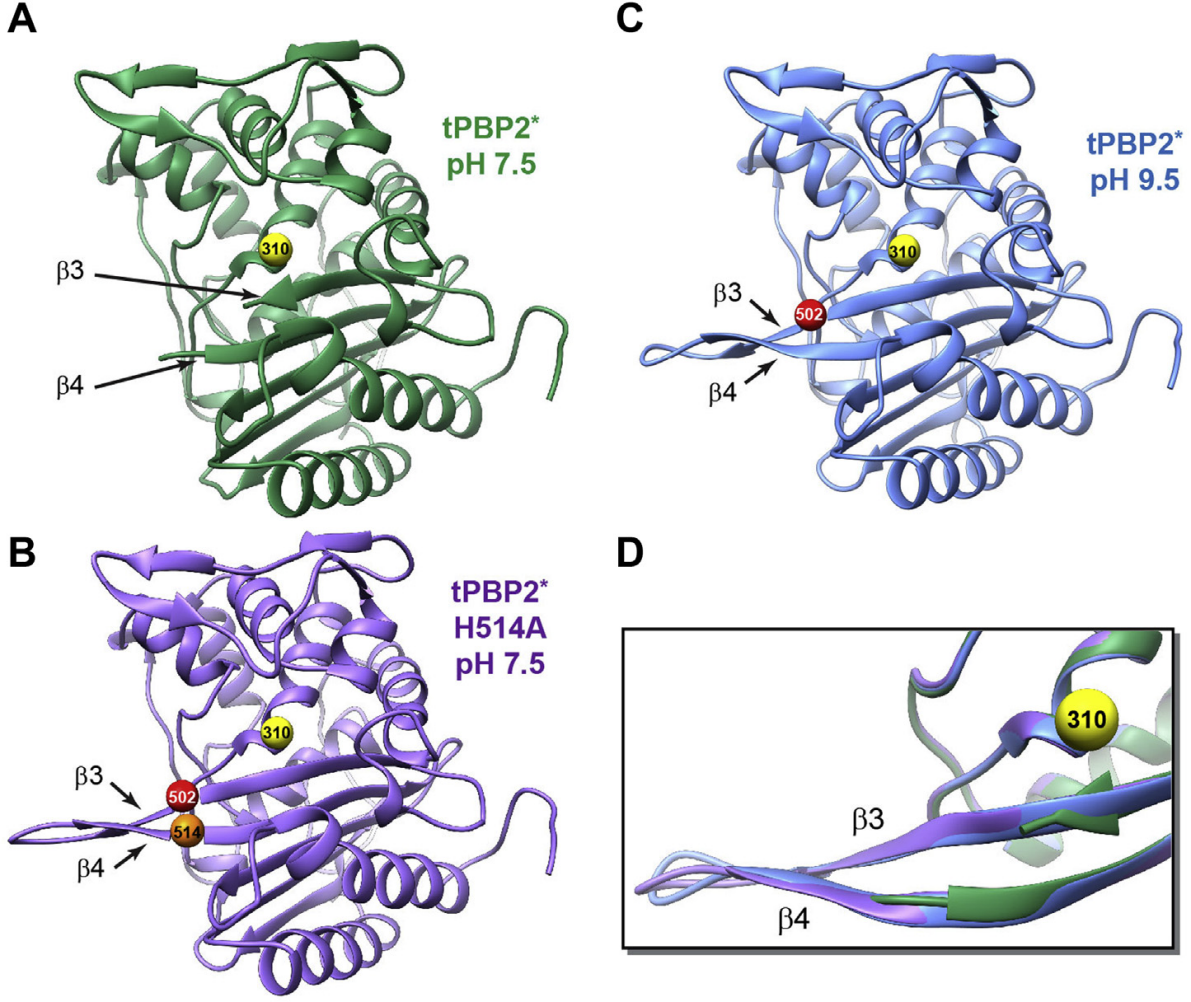
**Critical role of His514 in regulating the conformational dynamics of the β3–β4 loop.***A*, ribbon representation of the tPBP2∗ crystal structure at pH 7.5, showing an unstructured β3–β4 loop. *B*, ribbon representation of the tPBP2∗–H514A crystal structure at pH 7.5, showing the β3–β4 loop in an extended conformation. *C*, ribbon representation of the tPBP2∗ crystal structure at pH 9.5, showing an extended β3–β4 loop conformation. *D*, a close-up of an overlay of the crystal structures of tPBP2∗ at pH 7.5 (*green*) and pH 9.5 (*blue*) and tPBP2∗–H514A at pH 7.5 (*purple*). The positions of S310A (*yellow sphere*), Arg502 (*red sphere*), and H514A (*orange sphere*) are labeled. tPBP2, truncated construct of penicillin-binding protein 2.

**Figure S3:**
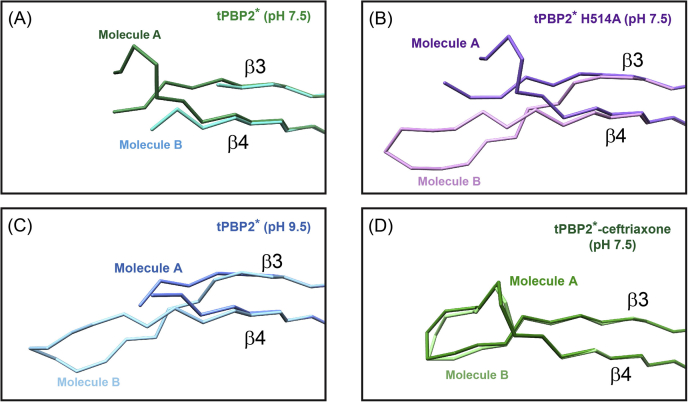
**Comparison of the β3–β4 loop conformations in molecules A and B of tPBP2∗ crystal structures.** Cα traces of the tPBP2∗ β3–β4 loop from residues 496 to 520 in tPBP2∗ structures are shown for (*A*) tPBP2∗ at pH 7.5 with molecule A in *green* and molecule B in *turquoise*, (*B*) tPBP2∗-H514A at pH 7.5 with molecule A in *purple* and molecule B in *pink*, (*C*) tPBP2∗ at pH 9.5 with molecule A in *dark blue* and molecule B in *light blue*, and (*D*) the tPBP2∗-ceftriaxone complex at pH 7.5, with molecule A in *dark green* and molecule B in *light green*.

**Figure S4:**
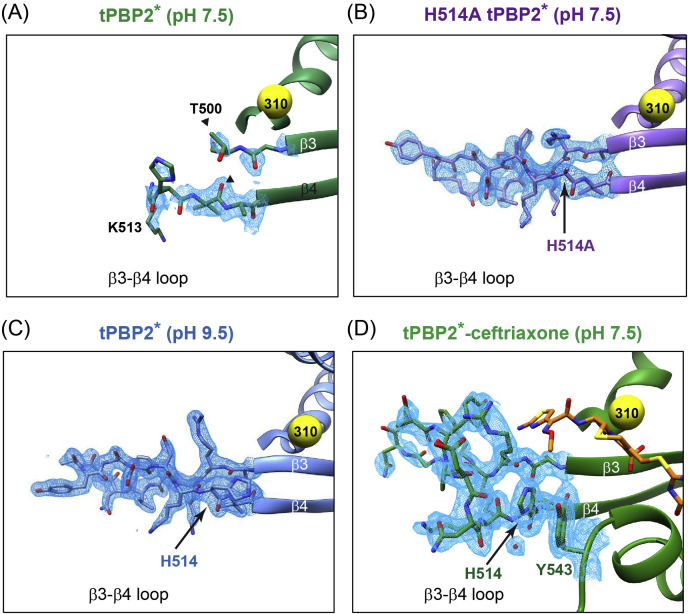
**Densities of the β3–β4 loop in different tPBP2∗ structures.** Omit (2mFo-DFc) maps contoured at 1σ for residues Ala501-Val515 of the β3–β4 loop in the crystal structures of tPBP2∗ at pH 7.5, tPBP2∗–H514A at pH 7.5, tPBP2∗ at pH 9.5, and the tPBP2∗–ceftriaxone complex at pH 7.5 are shown in *panels A*, *B*, *C*, and *D*, respectively. *Panel D* also includes the omit density for Tyr543, which interacts with His514, as well as the density for water molecules forming hydrogen bonds with His514 and the β3–β4 loop backbone. All conformations are from molecule B in the unit cells.

In the structure of tPBP2∗ determined at pH 7.5, the β3–β4 loop exhibits disorder in both molecules of the asymmetric unit but to different degrees (Fig. S3*A*). In molecule B, residues 501 to 514, a region spanning the β3–β4 loop, lack electron density, indicating disorder (Figs. 3*A* and S4*A*). Although the electron density is relatively weak, more residues could be modeled in molecule A, and it can be seen clearly that the loop adopts the inward and twisted conformation observed previously in PBP2 acylated by ESCs (17) (Fig. S3*A*). In the structure of tPBP2∗–H514A, also solved at pH 7.5, the β3–β4 loop of molecule A is similarly partially ordered, and where visible, it adopts the same inward and twisted conformation seen in the tPBP2∗ structure (Fig. S3*B*). By contrast, there is continuous electron density for the entire β3–β4 loop in molecule B, indicating that it is ordered, and it adopts an extended β-sheet conformation projecting away from the molecule (Figs. 3*B* and S4*B*). It therefore appears that mutating His514 to Ala increases the ordering of the β3–β4 loop in molecule B. In the structure of tPBP2∗ at pH 9.5, we also observed complete density for the β3–β4 loop in molecule B in the extended conformation (Figs. 3*C* and S4*C*). The backbone RMSD between the structures of tPBP2∗ at pH 9.5 and tPBP2∗–H514A in this region, comprising residues T498–I519, is 0.56 Å (Fig. 3*D*). Similar to both of the pH 7.5 structures, the β3–β4 loop of molecule A is partially ordered and adopts the inward and twisted conformation (Fig. S3*C*).

**Figure S5:**
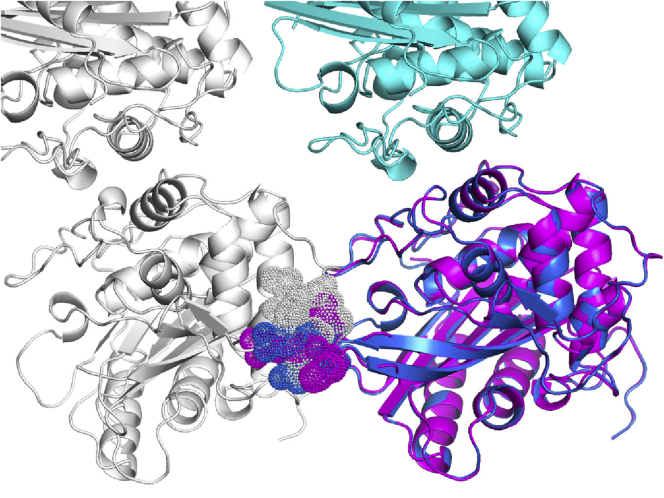
**Crystal packing selects against the extended β3–β4 conformation for molecule A in the P2**_**1**_**space group.***Gray* molecules are generated via symmetry expansion of the two tPBP2∗ molecules in the asymmetric unit from the structure of tPBP2∗ at pH 7.5. This figure was constructed by superimposing molecule B from the pH 9.5 structure (*blue*) and from the structure of the H514A mutant at pH 7.5 (*purple*) onto molecule A. van der Waals (vdW) clashes between residues of the extended β3–β4 loop (*blue* and *purple*) and those from the neighboring molecule (*gray*) are shown by overlapping colored *dots*. For clarity, the original molecule B (*cyan*) is also shown. vdW spheres are shown for all atoms of the clashing residues.

Examination of the packing environment around the β3–β4 loop of molecule A indicates there is insufficient space to accommodate an extended conformation (Fig. S5). By contrast, the β3–β4 loop of molecule B in several crystal structures of PBP2 is able to adopt different conformations. This is shown by (i) its transition from disordered to extended conformation in response to pH, (ii) its extended conformation in the H514A mutant, or (iii) its inward and twisted conformation when acylated by ceftriaxone (17). This suggests that the β3–β4 loop in molecule B in the crystal structures is more representative of the conformational behavior of this loop in solution. The extended β3–β4 loop conformations in molecule B have been observed previously in the structures of tPBP2^WT^ (Protein Data Bank [PDB]: 6P53) at pH 9.3 (17) and tPBP2∗ (PDB: 6VBM) at pH 9.3 (18), even though they were obtained using different crystallization conditions (Ches and PEG 600 *versus* PEG 400 and PEG 1500). These observations reinforce the notion that the extended conformation of the β3–β4 loop is stabilized by high pH and the H514A mutation, and that it correlates with the upfield signal of R502C_BTFA_. It is therefore also likely that the downfield shifted signal of R502C^BTFA^ correlates with the inward and twisted state captured in molecule A.

### Contributions of His514 to β3–β4 loop conformation and ceftriaxone binding

**Figure 4:**
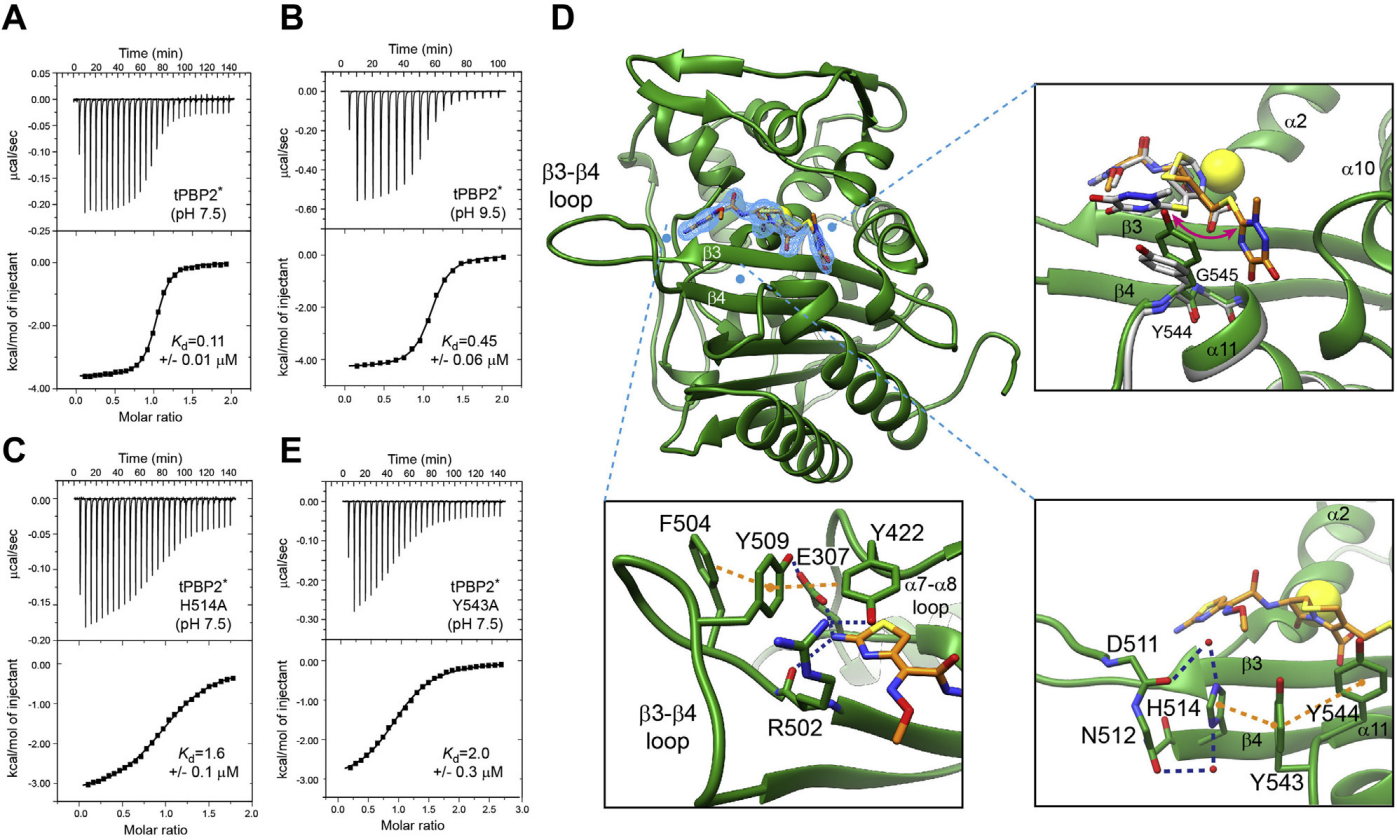
**Roles of H514 and Y543 in determining the β3–β4 conformation in ceftriaxone-bound tPBP2∗.** Isothermal titration calorimetry (ITC) measurements for ceftriaxone binding with tPBP2∗ at pH 7.5 and pH 9.5 are shown in panels (*A*) and (*B*), respectively. ITC measurements for ceftriaxone binding with tPBP2∗–H514A at pH 7.5 are shown in panel (*C*). *D*, ribbon representation of the crystal structure of the precovalent tPBP2∗–ceftriaxone complex. Ceftriaxone is represented as a stick model, with its omit density (2m*F*o–D*F*c) shown in a *blue mesh* contoured at 1σ, and S310A is shown as a *yellow sphere*. The *upper right inset* shows a close-up of the overlay of the precovalent ceftriaxone complex (*green model*) with the covalent acyl complex (Protein Data Bank: 6P54) (*gray model*) revealing a 90° rotation of the dioxotriazine group; the *lower left inset* shows a close-up view of the buried thiazole group in the precovalent ceftriaxone complex. Residues that form important interactions are shown as *sticks*, with π stacking depicted as *orange-dotted lines*, and hydrogen bonds depicted as *blue-dotted lines*. The *lower right inset* shows interactions of the His514 side chain in the precovalent ceftriaxone complex with Tyr543–Tyr544 and water-mediated hydrogen bonds with the backbone carbonyls of Asp511 and Asn512. *E*, ITC measurements for ceftriaxone binding with tPBP2∗–Y543A at pH 7.5. tPBP2, truncated construct of penicillin-binding protein 2.

After demonstrating by solution NMR that high pH or the H514A mutation shifts the conformational equilibrium of the β3–β4 loop toward an extended conformational state, we investigated the capacity of this state to bind ceftriaxone. For these experiments, we used the tPBP2∗ variant to measure the noncovalent binding affinity of ceftriaxone in the absence of acylation, as described previously (17). Isothermal titration calorimetry (ITC) measurements revealed a *K*_*d*_ value of 0.11 ± 0.01 μM for the binding affinity of ceftriaxone to tPBP2∗ at pH 7.5 (Fig. 4*A*). In comparison, increasing the pH to 9.5 reduced the affinity of ceftriaxone by approximately 4-fold (*K*_*d*_ = 0.45 ± 0.06 μM; Fig. 4*B*), whereas introduction of the H514A mutation to tPBP2∗ reduced the affinity by approximately 15-fold (*K*_*d*_ = 1.6 ± 0.1 μM; Fig. 4*C*). As both increasing pH and the H514A mutation favor the extended β3–β4 loop in solution, these results further support the notion that the extended β3–β4 loop has reduced binding affinity for ceftriaxone.

We have previously reported the crystal structure of the acylated tPBP2^WT^–ceftriaxone complex, which shows that His514 is not involved in any direct interaction with ceftriaxone (17). In order to verify that the reduced affinity between tPBP2∗–H514A and ceftriaxone is not because of a loss of a direct His514–ceftriaxone interaction prior to acylation, we determined the crystal structure of the noncovalent complex of tPBP2∗ with ceftriaxone at pH 7.5 (Table S1). tPBP2∗ crystallized in the same space group as the *apo* structures, and the precovalent complex was obtained by soaking crystals in a ceftriaxone solution prior to cryoprotection. One ceftriaxone molecule was observed in the active site of each of the two molecules of the asymmetric unit, and a third ceftriaxone molecule was found at the packing interface of the two molecules, away from the active site, as previously reported (12).

Clear electron density is observed for the ceftriaxone molecule in the active sites of both molecules in the asymmetric unit, with marginally better density in molecule B (Fig. 4*D*). The β-lactam ring is situated directly adjacent to where the serine nucleophile (Ser310) would normally be, as expected (Fig. 4*D*, *lower right panel*). The R1 aminothiazole ring inserts between the protein core and the side chain of Tyr422 of the loop following α8 to form parallel π–π stacking. The terminal amino group of the aminothiazole ring is capped by two hydrogen bonds: one with the side chain of Glu307, located in the loop preceding the α2 helix, and the other with the carbonyl oxygen of Arg502 (Fig. 4*D*, *bottom left panel*). The C3 dioxotriazine ring of ceftriaxone, which is normally released following bond rearrangement upon acylation, forms parallel π–π stacking with the side chain of Tyr544 of the β5–α11 loop and inserts between Tyr544 and the loop C-terminally to the α10 helix (Fig. 4*D*, *upper right panel*). Interestingly, its position is on the opposite side of Tyr544 when compared with a previous crystal structure of tPBP2^WT^ acylated by ceftriaxone in which this group was unexpectedly retained in the postcovalent state (17) (Fig. 4*D*, *upper right panel*). The different positions of the dioxotriazine ring in the noncovalent *versus* acylated complexes are likely the result of the opening of the β-lactam ring in the acylated structure and subsequent relief of its strained conformation. Finally, the β3–β4 loop adopts the inward twisted conformation oriented toward the active site (Figs. S3*D* and S4*D*), where it forms interactions around the aminothiazole ring of ceftriaxone (Fig. 4*D*), and its conformation is essentially the same in both molecules of the asymmetric unit (Fig. S3*D*). Tyr509 appears to play an important role in stabilizing the twisted loop conformation, as its hydroxyl group forms a hydrogen bond with Glu307 and its aromatic ring forms perpendicular π–π stacking with the side chain of Tyr422. The twisted loop conformation may be in addition stabilized by interactions of a perpendicular π–π stacking between Phe504 and Tyr509 and a hydrogen bond between Tyr422 and Arg502 (Fig. 4*D*, *lower left panel*). Overall, this mode of ceftriaxone interaction is similar to our previously reported structure of tPBP2^WT^ acylated by ceftriaxone (17), suggesting that the twisting and rolling of the β3–β4 loop is not a consequence of acylation but instead is stabilized by noncovalent binding.

**Figure S6:**
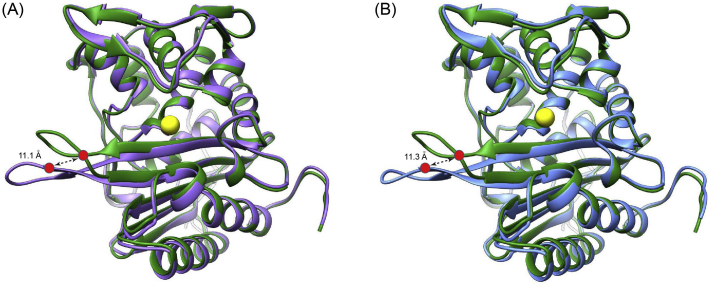
**Inward twisting of the β3–β4 loop upon ceftriaxone binding.** Superimposition of molecule B of the ceftriaxone–tPBP2∗ complex (*green*) with tPBP2∗–H514A at pH 7.5 (*purple*) or with tPBP2∗ at pH 9.5 (*blue*) are shown in *panels A* and *B*, respectively. Proteins are shown in the cartoon model. The location of S310A is labeled with a *yellow sphere*. The Cα atoms of residue Asp511 are labeled with a *red sphere*, and the distances of its movement in the overlaid structures are labeled.

Notably, in the noncovalent complex of tPBP2∗, His514 makes no contact with ceftriaxone. Instead, it forms a shifted π–π stacking interaction with Tyr543 located in the loop immediately preceding α11 (Fig. 4*D*, *right bottom panel*). Such an interaction might be enhanced by the positive charge of His514 at a lower pH, contributing an additional cation–π interaction (24). In addition, the His514 side chain forms two water-mediated hydrogen bonds with the backbone carbonyl oxygen atoms of Asp511 and Asn512, potentially orienting the β3–β4 loop toward ceftriaxone (Fig. 4*D*, *bottom right panel*). When comparing the ceftriaxone-bound structure of tPBP2∗ with the structures of tPBP2∗–H514A and tPBP2∗ at pH 9.5 in which the β3–β4 loop is extended, the Cα atoms of Asp511 are ∼11 Å apart (Fig. S6). These structural observations suggest an important role for His514 in the conformation of the β3–β4 loop and provide a molecular interpretation for the marked loss of ceftriaxone-binding affinity in the H514A mutant, despite a lack of direct interactions of His514 with ceftriaxone.

To assess the importance of the His514–Tyr543 stacking interaction, we measured the affinity of a tPBP2∗–Y543A mutant for ceftriaxone. Affinity was lowered by ∼20-fold compared with tPBP2∗ (*K*_*d*_ = 2.0 ± 0.3 μM; Fig. 4*E*), similar to the loss of binding affinity in the tPBP2∗–H514A mutant. These results suggest that His514 requires the interaction with Tyr543 to orient the β3–β4 loop toward the active site and thus enhance the noncovalent binding affinity of ceftriaxone.

### Role of H514 in the TPase activity of PBP2

The aforementioned data suggest that His514 plays an important role in the conformation of the β3–β4 loop and thereby affects antibiotic binding. As a β-lactam, ceftriaxone is an analog of the d-Ala-d-Ala C terminus of the peptidoglycan substrate, and His514 may therefore have an important role in supporting essential TPase activity. To examine this, we carried out allelic exchange of the WT *penA* gene encoding PBP2 with the *penA* gene encoding His514 mutants, such that only those mutants that retained sufficient TPase activity to support growth would be selected. The codon for His514 was randomized in the context of the *penA41* allele encoding PBP2^H041^ (to allow for selection of transformants), and the resulting PCR fragments were transformed into the antibiotic-susceptible recipient strain, FA19. Since *N. gonorrhoeae* is a naturally competent organism, the PCR fragments recombine into the genome and replace the WT *penA* allele, and those mutants producing a functional PBP2 enzyme can be selected on agar-containing ceftriaxone.

As shown in Table 1, using DNA containing a completely randomized codon-514, a large majority (27 of 29; 93%) of the strains had a *penA* allele with His at codon-514, strongly suggesting that His is preferred at this position. The other strains had a Leu codon or a Trp codon at position 514. To increase the chances of identifying transformants with a non-His codon at position 514, we used oligonucleotides that either eliminated C from the first base of the codon ((A/G/T)NN) or changed the codon to CN(A/G) to select for amino acids other than histidine. Using this strategy, we identified Gln as an additional amino acid capable of supporting growth. Overall, our data indicate a clear preference for His at position 514, but because three other amino acids were also identified at this position, a histidine at this position is not strictly essential for TPase function. Although we did not ascertain the fitness of the strains with a non-His514 codon, colonies showed no obvious changes in morphology or size.

### Resistance mutants favor the low-affinity ceftriaxone-binding state

We have previously identified multiple mutations in the *penA* gene from the ceftriaxone-resistant strain H041 that confer ceftriaxone resistance to the organism when introduced into the WT *penA* gene from the antibiotic-susceptible strain FA19 (15, 16). Mutations of particular interest among these are F504L and N512Y in the β3–β4 loop, which we have proposed hinder the dynamics of the β3–β4 loop based on crystal structures of the ceftriaxone-acylated tPBP2^H041^ (18), and the G545S mutation in the loop immediately preceding α11 that we believe restricts rotation of β3 and thus hinders formation of the oxyanion hole. In order to examine their contribution to ceftriaxone binding in the preacylation complex, we mutated these residues in tPBP2∗ and measured affinity of ceftriaxone using ITC.

**Figure 5:**
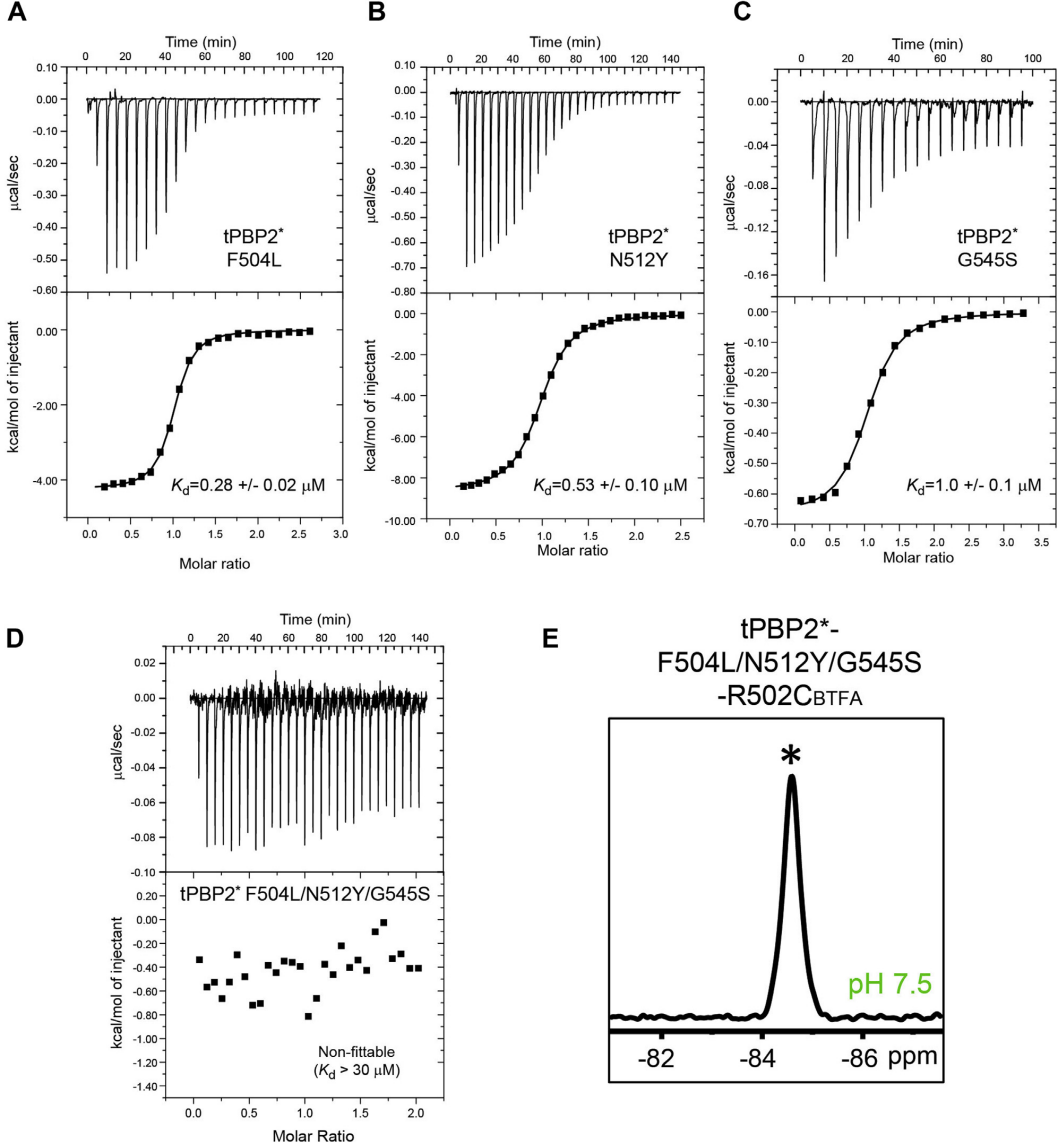
**Resistance mutations F504L, N512Y, and G545S reduce ceftriaxone binding and favor the extended β3–β4 loop conformation.** Isothermal titration calorimetry (ITC) measurements for ceftriaxone binding with tPBP2∗–F504L, tPBP2∗–N512Y, and tPBP2∗–G545S mutants are shown in panels (*A*), (*B*), and (*C*), respectively. *D*, ITC measurements for ceftriaxone with tPBP2∗–F504L/N512Y/G545S showing a nonfittable profile, indicating weak binding (estimated *K*_*d*_ > 30 μM based on a c value of 1). *E*, the ^19^F NMR spectrum of tPBP2∗–F504L/N512Y/G545S–R502C_BTFA_. The upfield peak position is labeled with an asterisk (∗). tPBP2, truncated construct of penicillin-binding protein 2.

Phe504, positioned at the N-terminal end of β3, does not interact with ceftriaxone in the ceftriaxone-bound preacylation complex but forms a perpendicular π–π stacking with Tyr509 in the inward conformation of β3–β4 (Fig. 4*D*, *lower left inset*). Mutation of Phe504 to Leu in tPBP2∗ (tPBP2∗–F504L) reduced the binding affinity for ceftriaxone by ∼2.6-fold compared with tPBP2∗ (Fig. 5*A*) (*K*_*d*_ = 0.28 ± 0.02 μM; Fig. 5*A*). Similarly, Asn512, which is located at the end of β4, also does not interact with ceftriaxone in the preacylation complex (Fig. 4*D*, *lower right inlet*), but its mutation to Tyr in tPBP2∗ again decreases ceftriaxone affinity, by 4.8-fold compared with tPBP2∗ (*K*_*d*_ = 0.53 ± 0.10 μM; Fig. 5*B*).

Of the three resistant mutations examined, G545S conferred the largest effect, with a 10-fold decrease in affinity of ceftriaxone (*K*_*d*_ = 1.0 ± 0.1 μM; Fig. 5*C*), which is on the same scale as that of the H514A (*K*_*d*_ = 1.6 ± 0.1 μM) or Y543A (*K*_*d*_ = 2.0 ± 0.3 μM) mutants (Fig. 4, *C* and *E*). In our structure of the noncovalent ceftriaxone complex of tPBP2∗, Gly545, which is located at the beginning of α11, does not form direct interactions with ceftriaxone (Fig. 4*D*, *upper right inlet*), and the same is true in the acylated complex of tPBP2^WT^ (17). By contrast, in the ceftriaxone-acylated tPBP2^H041^ complex, the side chain of Ser545 interacts with the carboxylate group of the β-lactam (18). This interaction is believed to be responsible for the markedly different binding mode observed for ceftriaxone in tPBP2^H041^ compared with tPBP2^WT^ and thus may explain the significantly reduced binding affinity of tPBP2∗–G545S for ceftriaxone (Fig. 5*C*). Importantly, the effects of these three mutations appear to be additive, as simultaneous mutation of all three residues in tPBP2∗–F504L/N512Y/G545S reduced the tPBP2∗ binding affinity by over 100-fold (estimated *K*_*d*_ > 30 μM based on the lower boundary of a fittable c value of 1 (Fig. 5*D*) (25)).

In order to investigate whether these mutations affect the conformational equilibrium of the two states observed in the *apo* protein, we recorded the ^19^F NMR spectrum of the tPBP2∗–F504L/N512Y/G545S–R502C_BTFA_ mutant. A single peak was observed at pH 7.5 compared with two ^19^F signals observed for tPBP2∗–R502C_BTFA_ (Fig. 5E). The chemical shift at 84.6 ppm aligns well with the upfield signal of both tPBP2-R502C_BTFA_ and tPBP2∗-R502C_BTFA_, the single signal of tPBP2–H514A–R502C_BTFA_ and tPBP2∗–H514A–R502C_BTFA_ (Fig. 2), and the single signal of tPBP2^H041^–R502C_BTFA_ (Fig. 1*C*). Given that the upfield signal is believed to correspond with the β3–β4 loop in the extended conformation (see aforementioned one), these data are consistent with the idea that the decreased binding affinity of the F504L/N512Y/G545S triple mutant for ceftriaxone is because the conformation of the β3–β4 loop is restricted to a single state, comprising the same extended conformation.

## Discussion

In this study, we examined the dynamic behavior of the β3–β4 loop of *N. gonorrhoeae* PBP2 using ^19^F-NMR, X-ray crystallography, and ITC. We provide evidence for distinct states of this loop in WT PBP2 in solution: one where the loop occupies an extended conformation and has a lower affinity for ceftriaxone, and one where the loop projects toward the active site (inward and twisted) and has a higher affinity. Shifts in pH alter the equilibrium between these states, and mutagenesis experiments suggest a key role for His514 in the conformational switching. We also show that PBP2 from the ESC-resistant strain H041 (tPBP2^H041^) occupies only a single state in solution, and that three mutations implicated in resistance, when incorporated into the WT protein, shift the equilibrium of the β3–β4 loop to the extended conformation and concomitantly reduce the affinity for ceftriaxone. These data are consistent with a mechanism where resistance mutations trap the β3–β4 loop in its extended and low-affinity state.

While the structures of *N. gonorrhoeae* PBP2 and various mutants have been studied extensively by crystallography (Table S2), its dynamics in solution has not been investigated until now. The β3–β4 loop of PBP2 was the focus of this study because it plays a critical role in acylation by ESCs (17), and mutations implicated in ESC resistance appear to hinder its movement during binding or acylation by ESCs (18). Specifically, crystal structures of tPBP2^WT^ have shown that the loop moves from the extended and outward conformation to the inward and twisted conformation when acylated by cefixime or ceftriaxone, whereas, in the crystal structure of tPBP2^H041^, it remains in an extended “outbent” conformation located relatively further away from the active site (18). However, since the β3–β4 loop is located on the surface of the protein where its structure may be influenced by crystal packing, whether such differences also occur in solution was unknown.

In accordance with the previous crystal structures, ^19^F-NMR spectra of tPBP2^WT^ revealed two distinct peaks, consistent with two conformations occurring in solution. We attribute these signals to the different conformational states of the β3–β4 loop because, when a residue on the β3–β4 loop is mutated (*i.e.*, His514 to Ala), only one peak is observed. This in turn suggests that His514 is required for the transition between the two states, and our observation that the equilibrium between the two states is altered by pH suggests that the protonation state of His514 is involved (see below).

Crystal structures of tPBP2∗ at pH 7.5 and 9.5 as well as of tPBP2∗–H514A and tPBP2∗–ceftriaxone complex at pH 7.5 suggest how pH alters the conformation of the β3–β4 loop. In molecule A of the asymmetric unit in all structures, the loop appears mostly disordered. The only density visible corresponds to the inward and twisted conformation, showing that the β3–β4 loop can occupy this conformation in the absence of ligands in the active site. Importantly, close crystal packing interactions around the loop in molecule A appear to preclude it from occupying the extended conformation (Fig. S5), whereas the environment around the loop in molecule B is more open and allows it to occupy different conformations. As a result, differences imparted by pH are reflected in molecule B of these structures. Consistent with a conformational equilibrium of states, the loop in molecule B is disordered at pH 7.5, but upon binding ceftriaxone, it transitions to the inward and twisted conformation as observed previously in acylated crystal structures of PBP2 (17). By contrast, in the *apo* structure at pH 9.5, the loop is highly ordered and exists in an extended conformation. Importantly, the loop is ordered in the structure of the H514A mutant, even though these data were collected from crystals buffered at pH 7.5. This suggests that His514 destabilizes the extended conformation of the β3–β4 loop at pH 7.5 but not at pH 9.5. These observations are nicely corroborated by the ^19^F-NMR solution studies because the upfield ^19^F signal corresponding to the extended state of the β3–β4 loop is predominant in the tPBP2–H514A mutant at pH 7.5, and the same is true at pH 9.5.

**Figure S7:**
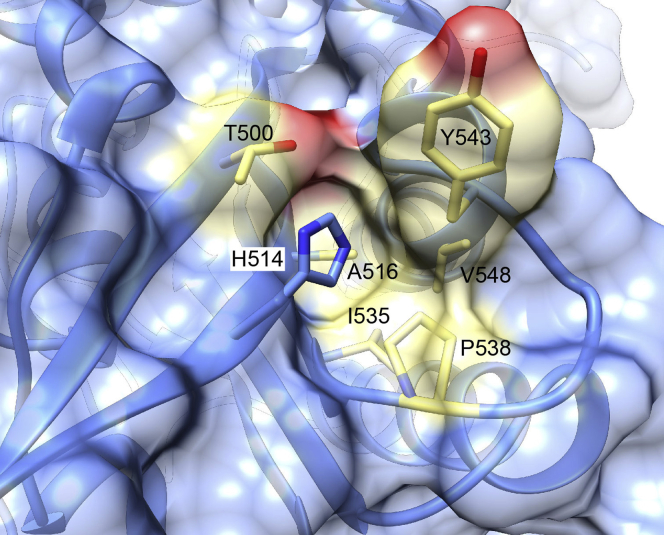
**The chemical environment of His514 in the extended conformation.** A *ribbon* representation of the tPBP2∗ structure at pH 9.5, with the β3–β4 loop in the extended conformation, is depicted in addition to a transparent surface representation (*blue*) of the region near His514. Hydrophobic regions are colored *yellow*, and contributing side chains are shown as *stick* models.

The central role for His514 in the conformational switching of the β3–β4 loop appears to be governed by its protonation state. Examination of the crystal structures suggests how increasing protonation of His514 would both stabilize the inward and twisted state and destabilize the extended conformation of the β3–β4 loop. In the extended β3–β4 loop conformation (*e.g.*, molecule B of tPBP2∗ at pH 9.5), the presumed deprotonated imidazole side chain of His514 forms a hydrogen bond with the carbonyl oxygen atom of Tyr543 but otherwise also points toward a predominantly hydrophobic pocket formed by Thr500, Ala516, Ile535, Pro538, Val548, and Tyr543 (Fig. S7). Packing within this environment would not be favored when His514 is protonated and charged and thus would destabilize the extended conformation of the β3–β4 loop. By contrast, the inward and twisted state (as seen in the tPBP2∗–ceftriaxone complex (Fig. 4*D*)) appears to be stabilized by His514 forming a shifted π–π stacking with the side chain of Tyr543, an interaction that would be enhanced by its protonation (24), and also by two water-mediated hydrogen bonds with the carbonyl groups of Asp511 and Asn512. Since *N. gonorrhoeae* colonizes different niches in the human host where pH varies, it is possible that deprotonation/protonation of His514 and subsequent alteration of the β3–β4 loop is a biological mechanism in response to differing environmental conditions.

Our data also have implications for understanding the molecular mechanism underpinning the ESC resistance of *N. gonorrhoeae*. From both X-ray crystallography and NMR investigations of tPBP2, it is very clear that the behavior of the β3–β4 loop is strikingly different in PBP2 variants derived from ESC-resistant strains. In the crystal structure of tPBP2^H041^, the loop occupies the extended outbent conformation (18), and ^19^F-NMR experiments in the present study show a single peak in the spectrum for tPBP2^H041^ rather than two for tPBP2^WT^. Moreover, when tPBP2^H041^ is acylated by ceftriaxone, the loop remains in the outbent conformation, and its movement therefore appears hindered (18).

**Figure S8:**
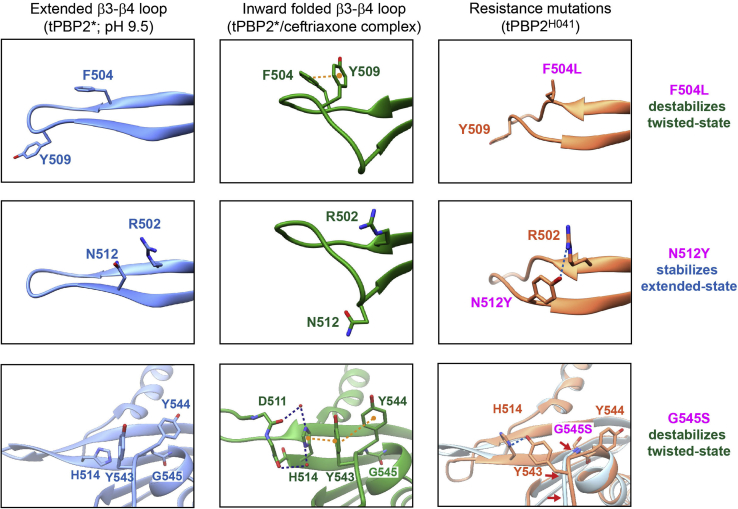
**Conformational effect of resistance-conferring mutations.** Mutation of F504L, N512Y, and G545S stabilizes the extended β3–β4 conformation or destabilizes the twisted β3–β4 conformation to generate ceftriaxone resistance. The pertinent residues in the extended β3–β4 loop and twisted β3–β4 loop of tPBP2∗ are shown in the *left* and *middle* panels, and H041 resistance mutations and their interacting residues are shown in the *right* panels. Shifts in α11 and its preceding loop associated with the G545S mutation in tPBP2^H041^ (*orange*) compared with tPBP2∗ (pH 9.5) are indicated by *red arrows*. Formations of favorable vdW interactions or hydrogen bonds are denoted by *dashed lines* in *orange* or *blue*, respectively. vdW, van der Waals.

Two mutations (F504L and N512Y) are present on the β3–β4 loop in PBP2^H041^ and, consistent with their role in ESC resistance, introduction into tPBP2^WT^ lowers the affinity of PBP2 for ceftriaxone by 2.6-fold for F504L and 4.8-fold for N512Y. Based on our current and previously published structures, we now understand how these mutations lower the capacity of the protein to be inhibited by ceftriaxone. In the noncovalent ceftriaxone-bound structure, Phe504 forms a perpendicular π–π stacking with Tyr509 in the inward and twisted conformation of the β3–β4 loop (Fig. S8), but this interaction is absent in the structure of tPBP2^H041^. Hence, by weakening this interaction, the F504L mutation may destabilize the high-affinity state for binding. In a similar manner, a hydrogen bond between the side chains of Arg502 and Tyr512 present in tPBP2^H041^ because of the N512Y mutation may stabilize the β3–β4 loop in its extended and low-affinity state, whereas these two residues are far apart in the inward and twisted β3–β4 loop as seen in the ceftriaxone-bound structure of tPBP2^WT^.

We also examined the effect on affinity of the G545S mutation observed in PBP2 from H041. In the crystal structure of tPBP2^H041^ acylated by ceftriaxone, the side-chain hydroxyl of Ser545 forms a hydrogen bond with the carboxylate group of ceftriaxone, and this interaction may be responsible for the altered binding mode of ceftriaxone observed in tPBP2^H041^ compared with that of tPBP2^WT^. Similar to mutations on the β3–β4 loop, introduction of G545S into WT PBP2 lowers the affinity for ceftriaxone by 10-fold. As G545S also shifts the backbone position of the loop harboring Tyr534 and Tyr544 (18), it may weaken the interaction between His514 and Tyr543, thus destabilizing the inward and twisted state of the β3–β4 loop.

**Figure 6:**
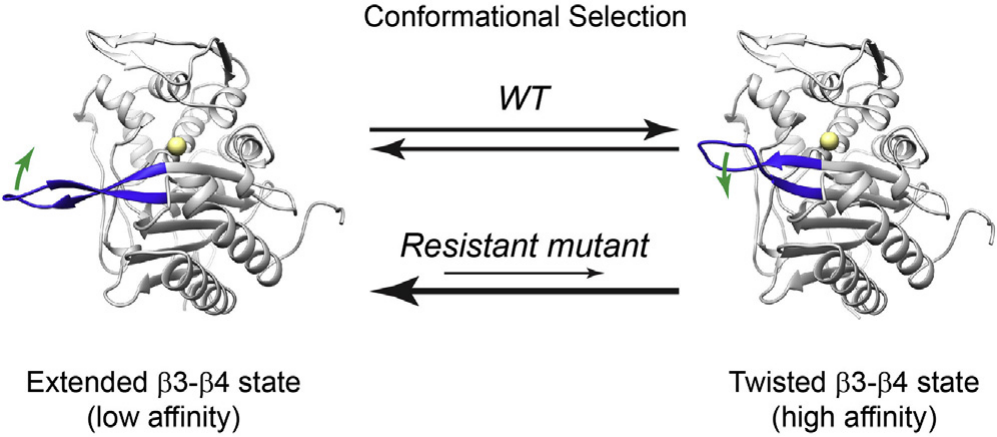
**Ceftriaxone resistance occurs by restricting the conformational access of a high-affinity binding state in PBP2.** PBP2 interconverts between two distinct conformational ensembles. The *left panel* depicts the low-affinity ceftriaxone-binding conformation with an extended β3–β4 loop (*blue cartoon structure*), whereas the *right panel* depicts the high-affinity ceftriaxone-binding conformation with a twisted β3–β4 loop. At neutral pH in the WT PBP2, the two states are found in a roughly equal ratio. Resistance-conferring mutations shift the equilibrium toward the low-affinity extended loop conformation. PBP2, penicillin-binding protein 2.

The overall picture emanating from our studies is of multiple conformations for the β3–β4 loop comprising low-affinity or high-affinity states for ceftriaxone binding (Fig. 6). When the loop is in the extended conformation, the affinity of PBP2 for ceftriaxone is lower, and when it occupies the inward and twisted conformation, its affinity for ceftriaxone is higher. It is also becoming clear that mutations associated with ESC resistance shift the equilibrium of the loop toward an extended and low-affinity state, thereby hindering the conformational change in the β3–β4 loop that is necessary to form the higher-affinity state. These data also address the question of whether the inward and twisted state of the β3–β4 loop results from induced fit after binding of ligand or by conformational selection. Previously, the inward and twisted state was only observed in acylated structures of PBP2, but its presence at lower occupancy in *apo* crystal structures of PBP2 is direct evidence of conformational selection being the prevailing mechanism of binding.

Our findings are highly reminiscent to those reported recently for the Abl kinase, where the kinase domain interconverts between one active and two inactive conformational states, and mutations confer resistance by depleting the conformation to which imatinib binds (26). Alteration of protein dynamics as a mechanism of drug resistance has also been documented in a number of other systems, including HIV-1 protease (27) and reverse transcriptase (28), retroviral integrase (29), and *Mycobacterium tuberculosis* InhA (30). A detailed understanding of such indirect resistance mechanisms that alter protein dynamics and conformational equilibria may facilitate the development of novel therapeutics.

Finally, our results have implications for understanding the essential function of PBP2 in peptidoglycan synthesis because the mechanism underlying the transition from a low-affinity state to a high-affinity state has presumably evolved to enhance TPase activity. Since the extended conformation of the β3–β4 loop clearly exists, it may serve to load the peptidyl substrate onto PBP2 prior to folding inward toward the active site, thus positioning the penultimate d-Ala for nucleophilic attack by Ser310. Transitioning between open and closed states of the β3–β4 loop may therefore be a means to accommodate the substrate, which is considerably larger than a cephalosporin antibiotic and requires a large binding surface area. Understanding the nature of PBP2–peptidoglycan interactions will address this question.

## Experimental procedures

### Cloning and expression of tPBP2

For these studies, we used a truncated construct of PBP2, termed tPBP2, comprised of amino acids 237 to 581 of the PBP2 gene corresponding to the TPase domain (17, 20). In addition, residues 283 to 297 were omitted from the construct because these form a loop that projects away from the TPase domain that could hinder crystallization (19) and were replaced by two intervening glycines. The TPase constructs exhibit the same levels of penicillin acylation activity as the full-length protein (20). tPBP2 was inserted into a modified pET24b vector as a C-terminal fusion with His_6_-SUMO protein *via* In-Fusion cloning (Takara Bio) (31, 32). A triple aspartate linker separates the C terminus of SUMO from the N terminus of tPBP2 (31, 32). The final expression construct contains His_6_-SUMO-DDD-tPBP2. After verification by DNA sequencing, the plasmids were transformed into BL21 STAR (DE3) competent *Escherichia coli* cells (Thermo Fisher Scientific) for protein expression. Generation of the various mutants described was carried out using QuikChange site-directed mutagenesis (Agilent). Plasmid DNA was sequenced to verify the presence of the desired mutation.

BL21∗ (DE3) cells containing the plasmids for the tPBP2 constructs were grown in M9 media at 37 °C until an absorbance of 0.6 at 600 nm was reached and then induced with 0.2 mM IPTG at 20 °C for 16 h. Cultures were harvested by centrifugation, resuspended in a lysis buffer containing 25 mM Tris (pH 7.5) and 500 mM NaCl (hereafter referred to as “lysis buffer”), and lysed using a French press. The lysates were then clarified by centrifugation at 20,000*g* for 30 min.

### Purification of tPBP2 variants

All purification steps were carried out at 4 °C or on ice. For all protein purifications, the lysate supernatant was incubated with 10 ml of pre-equilibrated TALON metal affinity resin (Takara Bio). The resin was washed with 100 ml of lysis buffer containing 5 mM imidazole, and tPBP2 was eluted with 50 ml of lysis buffer containing 200 mM imidazole.

For crystallography and ITC studies, protein purification proceeded as described previously, with a subsequent digestion step. After elution from the TALON resin, the protein sample was diluted with buffer and concentrated several times in lysis buffer, resulting in a 100-fold overall dilution of imidazole, and then concentrated to a final volume of 25 ml. DTT (0.5 mM) and a 1:100 M ratio of His-tagged SUMO protease:SUMO-tPBP2 fusion protein were added, and the sample was incubated with shaking at 4 °C overnight. After digestion, the sample was diluted to 50 ml with lysis buffer and passed over a 10-ml pre-equilibrated HisPur nickel–nitrilotriacetic acid (Thermo Fisher Scientific) column, and the column was washed with 100 ml of lysis buffer containing 20 mM imidazole. The flow through and wash fractions were analyzed by SDS-PAGE to identify fractions containing the digested protein. Uncleaved fusion protein, cleaved His_6_-SUMO, and His_6_-SUMO protease remained bound to the nickel–nitrilotriacetic acid column during the washes and were removed using 50 ml of lysis buffer with 250 mM imidazole. Flow through and wash fractions were pooled and concentrated to 2 ml and purified to homogeneity using a Superdex 200 size-exclusion column (GE Healthcare Life Sciences) run in the final buffer: 25 mM Tris (pH 7.5) and 100 mM KCl.

### Solution NMR measurements

For solution NMR studies, proteins were purified as described previously except that SUMO digestion was not performed. After elution in 50 ml of lysis buffer with 200 mM imidazole, 1 mM Tris(2-carboxyethyl)phosphine and a 10:1 M ratio of BTFA:protein were added to the sample. After 1 h of rocking at 4 °C, the sample was buffer exchanged 10-fold into NMR buffer (25 mM Tris, pH 7.5, and 100 mM KCl) and concentrated to 2 ml. The sample was passed through a PD-10 column (GE), which had been pre-equilibrated with NMR buffer, and elution fractions containing protein were pooled. BTFA-labeling efficiency was evaluated using MeO-PEG-5000-maleimide. Labeled protein was incubated with a 10-fold molar excess of the PEG–maleimide for 1 h at room temperature, after which samples were analyzed using SDS-PAGE. The maleimide moiety binds to free cysteine thiols unmodified by BTFA and causes a mobility shift of 5000 Da on SDS-PAGE. BTFA alkylation of cysteines followed by PEG maleimide incubation did not result in a mobility shift on SDS-PAGE, indicating that the BTFA reaction was at least 95% complete. If necessary, samples were further buffer exchanged 50-fold into tri-buffer at various pH values. The tri-buffer contains 25 mM Tris, 25 mM Bis–Tris, 50 mM sodium acetate, and 100 mM KCl and has a buffering range of pH 4 to 10.

Protein samples for NMR were concentrated to 100 μM and loaded into a 5-mm NMR tube with 10% heavy water as the lock signal. ^19^F spectra were recorded at 15 °C on a Bruker 500 MHz spectrometer with a ^19^F cryoprobe. NMRPipe (33) was used to process NMR data, and iNMR was used to generate figures (www.inmr.net).

### Crystallization of tPBP2∗

Protein samples were concentrated to 10 mg/ml, and crystal trays were set up using the sitting drop vapor diffusion method, with 1 μl of protein and 1 μl of well solution combined in each drop. The commercially available sparse matrix screens JCSG Cores I-IV and PEGs I+II (QIAGEN) were used for initial screening at 20 °C. Crystallization “hits” were then optimized by microseeding and varying precipitant and salt concentrations. The neutral pH tPBP2∗ crystals were obtained in 30% PEG 400 and 0.1 M Hepes (pH 7.5). The high pH tPBP2∗ crystals were obtained in 26% PEG 1500, 25 mM Tris, 25 mM Bis–Tris, and 50 mM sodium acetate (pH 9.5), mirroring the condition of the NMR sample. H514A tPBP2∗ crystals were from a condition containing 30% PEG 1500 and 0.1 M Tris at pH 7.5.

Crystals were cryoprotected in well solution containing 35% ethylene glycol and flash frozen in liquid nitrogen. The complex with ceftriaxone was obtained by soaking tPBP2∗ crystals obtained in 22% PEG 3350 and 0.15 M potassium sulfate in a solution of 10 mM ceftriaxone, 22% PEG 3350, and 0.15 M potassium sulfate for 30 min, followed by cryoprotection with 35% ethylene glycol and 5 mM ceftriaxone in the same well solution.

### Structural analysis of tPBP2∗ and the ceftriaxone complex

Diffraction data were collected at the SER-CAT 22-ID and 22-BM beamlines and NE-CAT 24-IDC and 24-IDE beamlines and processed using XDS (34). Crystal structures were solved using molecular replacement in the PHASER module of the PHENIX suite (35), using PDB ID code 6P53 (17) as the search model. Coot (36) and PHENIX (35) were used for iterative model building and refinement, and eLBOW (37) was used to generate restraints for ceftriaxone. Composite omit maps were generated using PHENIX (35). Notably, molecule A of the *apo* structures of tPBP2∗ and tPBP2∗–H514A at pH 7.5 (but not at pH 9.5) contains a bound sulfate or phosphate molecule adjacent to Thr498 of the KTG motif in the active site, similar to a phosphate group that was observed previously in the active site of tPBP2^WT^ (17). The origin of this ligand is unclear because neither sulfate nor phosphate was used during protein purification or crystallization, but the electron density is clearly tetrahedral in shape.

### Ceftriaxone-binding affinity measurements

A MicroCal VP-ITC instrument was used to carry out ITC measurements. Experiments were performed at 25 °C with 30 μM tPBP2∗ in the cell and 300 to 500 μM ceftriaxone in the syringe. Experiments were conducted using a 300-rpm stirring rate, with a total of 27 injections of 10 μl of ceftriaxone into an initial volume of 1.4 ml of tPBP2∗. For WT, H514A, Y543A, and H514Y tPBP2∗, three technical replicates were conducted. For F504L, N512Y, G545S, and F504L/N512Y/G545S tPBP2∗, two technical replicates were conducted. Average and standard error are reported. Both ceftriaxone and PBP2 were in a buffer containing 25 mM Tris at the desired pH values (7.5 or 9.5) and 100 mM KCl. Data were analyzed using Microcal Origin 7 software.

## Data availability

The coordinates of tPBP2∗–H514A (pH 7.5), tPBP2∗ (pH 7.5), tPBP2∗ (pH 9.5), and the tPBP2∗–ceftriaxone complex (pH 7.5) have been deposited to the PDB with accession codes of 6XQX, 6XQZ, 6XQY, and 6XQV, respectively.

## Supporting information

This article contains supporting information.

## Conflict of interest

The authors declare that they have no conflicts of interest with the contents of this article.
