# Supplementary material for: Mutations in PBP2 from ceftriaxone-resistant Neisseria gonorrhoeae alter the dynamics of the β3–β4 loop to favor a low-affinity drug-binding state
Source: J Biol Chem. 2021 Sep 13;297(4):101188. doi: 10.1016/j.jbc.2021.101188 (PMC8503634; doi:10.1016/j.jbc.2021.101188)
Supplement: Tables S1 and S2 [file mmc1.docx]

**Supplementary Table S1:** **Data collection and statistics of the tPBP2^*^_NG_ structures**

|  | **tPBP2*-H514A**  **(pH 7.5)** | **tPBP2***  **(pH 7.5)** | **tPBP2***  **(pH 9.5)** | **tPBP2*/ceftriaxone**  **(pH 7.5)** |
| --- | --- | --- | --- | --- |
| **PDB** | **6XQX** | **6XQZ** | **6XQY** | **6XQV** |
| **Data collection** |  |  |  |  |
| Wavelength (Å) | 1.0000 | 1.0000 | 0.9792 | 0.9795 |
| Space group | P 1 21 1 | P 1 21 1 | P 1 21 1 | P 1 21 1 |
| Cell dimensions |  |  |  |  |
| *a*, *b*, *c* (Å) | 44.5, 77.3, 88.2 | 42.5, 77.4, 86.7 | 44.9, 76.5, 87.9 | 42.6, 77.9, 87.7 |
| α, β, γ (°) | 90, 91.6, 90 | 90, 91.9, 90 | 90, 91.6, 90 | 90, 91.1, 90 |
| Resolution (Å) | 44.50-2.15 (2.23-2.15) | 42.52 – 2.04 (2.11 – 2.04) | 44.92 – 1.90 (1.97-1.90) | 43.84-2.05 (2.10-2.05) |
| *R*_meas_ | 0.133 (0.389) | 0.069 (0.394) | 0.052 (0.661) | 0.109 (0.609) |
| Mean *I*/σ*I* | 7.07 (3.16) | 16.47 (4.09) | 17.6 (2.6) | 11.80 (3.33) |
| Completeness (%) | 95.6 (97.9) | 98.0 (96.9) | 99.3 (99.5) | 99.5 (99.6) |
| Redundancy | 5.6 (5.8) | 4.2 (4.2) | 4.8 (4.8) | 5.6 (5.2) |
| Total reflections | 173966 (18740) | 147783 (14375) | 222043 (22545) | 200265 (18736) |
| Unique reflections | 31251 (3231) | 35213 (3454) | 46696 (4690) | 35913 (3586) |
|  |  |  |  |  |
| **Refinement** |  |  |  |  |
| *R*_work_ / *R*_free_ | 0.216/0.243 | 0.164/0.213 | 0.165/0.195 | 0.168/0.203 |
| No. atoms | 5044 | 5199 | 5091 | 5429 |
| Protein | 4949 | 4902 | 4948 | 5075 |
| Ligand/ion | 25 | 27 | 1 | 124 |
| Water | 70 | 270 | 142 | 230 |
| Average *B*-factors | 59.1 | 32.3 | 47.0 | 33.4 |
| Protein | 59.1 | 32.0 | 47.0 | 32.7 |
| Ligand/ion | 73.5 | 49.2 | 76.0 | 51.6 |
| Water | 50.2 | 35.5 | 47.1 | 39.2 |
| R.m.s. deviations |  |  |  |  |
| Bond lengths (Å) | 0.002 | 0.008 | 0.007 | 0.005 |
| Bond angles (°) | 0.62 | 0.95 | 0.90 | 0.96 |
| Ramachandran |  |  |  |  |
| Favored (%) | 98.60 | 97.93 | 98.74 | 98.15 |
| Allowed (%) | 1.40 | 2.07 | 1.10 | 1.54 |
| Outliers (%) | 0.00 | 0.00 | 0.16 | 0.31 |

*Values in parentheses are for highest-resolution shell.

**Supplementary Table S2:** **Crystal structures of *N. gonorrhoeae* penicillin-binding protein 2**

| **Structure** | **PDB** | **Reference** |
| --- | --- | --- |
| *Structures solved using full-length construct:* | | |
| Wild-type in *apo* form | 3EQU | Powell *et al.* (2009). J. Biol. Chem 284, 1202-1212 |
| PBP2 containing 4 mutations associated with penicillin resistance | 3EQZ | Powell *et al.* (2009). J. Biol. Chem 284, 1202-1212 |
| PBP2 containing an A501T mutation | 5KSH | Tomberg *et al.* (2017) Biochem. 56, 1140-1150 |
|  |  |  |
| *Structures solved using transpeptidase domain construct:* | | |
| Wild-type in *apo* form | 6P53 | Singh *et al.* (2019) J. Biol. Chem. 294, 14020-14032 |
| Wild-type with bound phosphate in the active site | 6P52 | Singh *et al*. (2019) J. Biol. Chem. 294, 14020-14032 |
| Wild-type acylated by ceftriaxone | 6P54 | Singh *et al.* (2019) J. Biol. Chem. 294, 14020-14032 |
| Wild-type acylated by cefixime | 6P55 | Singh *et al.* (2019) J. Biol. Chem. 294, 14020-14032 |
| T498A mutant of wild-type in *apo* form | 6P56 | Singh *et al.* (2019) J. Biol. Chem. 294, 14020-14032 |
| S310A mutant of wild-type in *apo* form | 6VBM | Singh *et al*. (2020). J. Biol. Chem. 295, 7529-7543 |
| S310A mutant of wild-type in *apo* form at pH 7.5 | 6XQZ | This study |
| S310A mutant of wild-type in *apo* form at pH 9.5 | 6XQY | This study |
| S310A/H514A mutant of wild-type in *apo* form | 6XQX | This study |
| S310A mutant of wild-type in noncovalent complex with ceftriaxone | 6XQV | This study |
| PBP2 from the penicillin-resistant strain 6140 | 4U3T | Fedarovich *et al.* (2014) Biochem. 53, 7596-7603 |
| PBP2 from the ESC reduced susceptibility strain 35/02 in *apo* form | 6VBL | Singh *et al.* (2020). J. Biol. Chem. 295, 7529-7543 |
| PBP2 from the ESC-resistant strain H041 in *apo* form | 6VBC | Singh *et al.* (2020). J. Biol. Chem. 295, 7529-7543 |
| PBP2 from the ESC-resistant strain H041 acylated by ceftriaxone | 6VBD | Singh *et al.* (2020). J. Biol. Chem. 295, 7529-7543 |
